# Flavonoid Extract of *Senecio scandens* Buch.-Ham. Ameliorates CTX-Induced Immunosuppression and Intestinal Damage via Activating the MyD88-Mediated Nuclear Factor-κB Signaling Pathway

**DOI:** 10.3390/nu17152540

**Published:** 2025-08-01

**Authors:** Xiaolin Zhu, Lulu Zhang, Xuan Ni, Jian Guo, Yizhuo Fang, Jianghan Xu, Zhuo Chen, Zhihui Hao

**Affiliations:** 1 Innovation Centre of Chinese Veterinary Medicine, College of Veterinary Medicine, China Agricultural University, Beijing 100193, China; zhuxiaolin1103@163.com (X.Z.); zhanglulu@cau.edu.cn (L.Z.); nixuandream@outlook.com (X.N.); guojiancloud@sina.com (J.G.); fyz980088310@gmail.com (Y.F.); 15750510829@163.com (J.X.); 2 State Key Laboratory of Veterinary Public Health and Safety, College of Veterinary Medicine, China Agricultural University, Beijing 100193, China; 3 Key Biology Laboratory of Chinese Veterinary Medicine, Ministry of Agriculture and Rural Affairs, Beijing 100193, China

**Keywords:** *Senecio scandens* flavonoids, cyclophosphamide, immunomodulation, NF-κB signaling pathway, intestinal damage, gut microbiota

## Abstract

**Background/Objectives:** *Senecio scandens* Buch.-Ham. is a flavonoid-rich traditional medicinal plant with established immunomodulatory properties. However, the mechanisms underlying the immunoregulatory and intestinal protective effects of its flavonoid extract (*Senecio scandens* flavonoids—SSF) remain unclear. This study characterized SSF’s bioactive components and evaluated its efficacy against cyclophosphamide (CTX)-induced immunosuppression and intestinal injury. **Methods:** The constituents of SSF were identified using UHPLC/Q-Orbitrap/HRMS. Mice with CTX-induced immunosuppression were treated with SSF (80, 160, 320 mg/kg) for seven days. Immune parameters (organ indices, lymphocyte proliferation, cytokine, and immunoglobulin levels) and gut barrier integrity markers (ZO-1, Occludin, Claudin-1 protein expression; sIgA secretion; microbiota composition) were assessed. Network pharmacology combined with functional assays elucidated the underlying regulatory mechanisms. **Results:** Twenty flavonoids were identified in SSF, with six prototype compounds detectable in the blood. The SSF treatment significantly ameliorated CTX-induced weight loss and atrophy of the thymus and spleen. It enhanced splenic T- and B-lymphocyte proliferation by 43.6% and 29.7%, respectively; normalized the CD4^+^/CD8^+^ ratio (1.57-fold increase); and elevated levels of IL-2, IL-6, IL-10, TNF-α, IFN-γ, IgM, and IgG. Moreover, SSF reinforced the intestinal barrier by upregulating tight junction protein expression and sIgA levels while modulating the gut microbiota, enriching beneficial taxa (e.g., the *Lachnospiraceae_NK4A136_group*, *Akkermansia*) and suppressing pathogenic *Alistipes*. Mechanistically, SSF activated the TLR/MyD88/NF-κB pathway, with isoquercitrin identified as a pivotal bioactive constituent. **Conclusions:** SSF effectively mitigates CTX-induced immunosuppression and intestinal damage. These findings highlight SSF’s potential as a dual-functional natural agent for immunomodulation and intestinal protection. Subsequent research should validate isoquercitrin’s molecular targets and assess SSF’s clinical efficacy.

## 1. Introduction

Immune homeostasis serves as the foundation for host defense and physiological equilibrium [1]. However, immunosuppression triggered by pathological conditions or pharmacological interventions disrupts this balance, increasing the risk of infection and cancer progression [2]. Epidemiological data indicate that the prevalence rate of immunosuppression reaches 6.6% among U.S. adults. This condition is particularly common in patients undergoing chemotherapy or organ transplantation, underscoring its significance as a public health concern [3]. Cyclophosphamide (CTX), a classic alkylating antineoplastic agent, exerts its cytotoxic effects by disrupting DNA replication and transcription [4]. While effectively inhibiting tumor growth, CTX concurrently induces severe immunosuppression and intestinal barrier damage [5,6]. Emerging evidence suggests that gut microbiota dysbiosis and impaired short-chain fatty acid (SCFA) metabolism are potential underlying mechanisms for these adverse effects. Recent research reveals a complex bidirectional regulatory network between the gut microbiota and the immune system [7,8,9]. Consequently, interventions targeting the gut microbiota/immune system axis represent a promising strategy for counteracting CTX-induced toxicity.

Natural products have garnered considerable attention in immunomodulatory drug discovery due to their structural diversity and multi-target regulatory properties [10]. *Senecio scandens* Buch.-Ham., a medicinal plant of the Asteraceae family, is officially listed in the Chinese Pharmacopoeia (2020 edition), with its dried aerial parts rich in bioactive constituents, including flavonoids (e.g., quercetin and kaempferol derivatives), alkaloids, and polysaccharides. Pharmacological investigations demonstrate that *Senecio scandens* extracts exhibit notable anti-inflammatory, antibacterial, antioxidant, antitumor, and immunomodulatory activities. [11,12,13]. For instance, *Senecio scandens* polysaccharides alleviate atopic dermatitis by modulating the gut microbiota and the MAPK/NF-κB pathway, while also stimulating nitric oxide (NO) production, the secretion of immunomodulatory cytokines (IL-1β and TNF-α), and splenocyte proliferation, which indicates potential immunostimulatory properties [14,15]. Notably, plant-derived secondary metabolites such as flavonoids possess broad immunomodulatory potential, including the regulation of Th1/Th2 balance, the activation of macrophage phagocytic functions, and the modulation of signaling pathways such as TLRs/MyD88/NF-κB [16,17,18]. However, despite preliminary evidence supporting the immunomodulatory activity of *Senecio scandens*, existing studies predominantly focus on polysaccharides or crude extracts. The mechanistic understanding of its core flavonoid fraction remains limited. The dual regulatory roles of *Senecio scandens* flavonoids (SSF), encompassing both systemic immunity and intestinal barrier repair, remain undefined. Key bioactive compounds and their mechanistic pathways remain unidentified. Furthermore, the interplay between SSF-driven gut microbiota remodeling and immune restoration has yet to be explored. Addressing these gaps will facilitate the transition of SSF from an empirical herbal remedy to a mechanism-defined functional product.

Therefore, we employed an integrated strategy to bridge these gaps. The experimental flowchart is presented in Supplementary Figure S1. A CTX-induced immunosuppression mouse model was established to comprehensively evaluate the immunomodulatory and intestinal protective effects of SSF. Serum pharmacochemistry was utilized to track the in vivo behavior of bioactive flavonoids. 16S rRNA sequencing was integrated to elucidate causal relationships between the gut microbiome and immune homeostasis. What is more, network pharmacology analyses, followed by in vitro and in vivo experimental validation, were employed to identify the TLR/MyD88/NF-κB signaling pathway as a key effector mechanism of SSF action. Our findings provide scientific evidence supporting the therapeutic potential of SSF and offer a theoretical foundation for its development as a functional food ingredient or feed additive.

## 2. Materials and Methods

### 2.1. Reagents and Materials

The aerial parts of *Senecio scandens* (origin: Shaanxi, China) were provided and authenticated by Prof. Yulin Lin from the Institute of Medicinal Plant Development, Chinese Academy of Medical Sciences. AB-8 macroporous adsorption resin was sourced from Nankai University Chemical Plant, China. The following reagents were obtained commercially: adonifoline (National Institutes for Food and Drug Control, Beijing, China); cyclophosphamide (CTX), levamisole hydrochloride (LH), hyperoside (≥98%), scutellarin (≥98%), quercetin-3β-D-glucoside (≥98%), rutin (≥98%), kaempferol (≥98%), kaempferol-3-O-rutinoside (≥98%), quercetin (≥98%), and afzelin (≥98%) (Yuanye Bio-Technology, Shanghai, China); lipopolysaccharide (LPS, Sigma-Aldrich, St. Louis, MO, USA); and concanavalin A (ConA), red blood cell lysis buffer, and BCA protein assay kit (Solarbio, Beijing, China). The fluorescence-labeled antibodies CD45-FITC, CD3-APC, CD4-PE, and CD8-PerCP were purchased from BioLegend (San Diego, CA, USA). ELISA kits for cytokines and immunoglobulins (IL-2, IL-6, IL-10, TNF-α, IFN-γ, IgG, IgM, sIgA) were acquired from Elabscience (Wuhan, China). The Cell Counting Kit-8 (CCK-8) was purchased from Selleck Chemicals (Shanghai, China), while coelenterazine was obtained from Bide Pharmatech (Shanghai, China). Primary antibodies, including anti-Phospho-NF-κB p65 (p-p65), anti-Phospho-IκBα (p-IκBα), and anti-Phospho-IKKα/β (p-IKK) were sourced from Cell Signaling Technology (Danvers, MA, USA); ZO-1, Occludin, Claudin-1, and GAPDH from Proteintech (Wuhan, China); and HRP-conjugated secondary antibody from Abbkine (Shanghai, China).

### 2.2. Preparation and Component Analysis of SSF

The dried whole plant of *Senecio scandens* was crushed and sieved to obtain a powder. The powder underwent triple extraction using 10 volumes of 60% ethanol under reflux (1 h per cycle). The combined extracts were filtered and centrifuged (5000 rpm, 10 min), and the supernatant was collected. After concentration under reduced pressure, the extract was reconstituted in water to a final concentration of 50 mg/mL (*w*/*v*). The solution’s pH was adjusted to 4–5 and then loaded onto an AB-8 macroporous adsorption resin column. Initial elution with water removed impurities, followed by desorption of total flavonoids using 95% (*v*/*v*) ethanol. The resulting eluate was concentrated under reduced pressure at 50–60 °C and subsequently lyophilized. This process yielded SSF as a yellowish-brown powder with an approximate recovery rate of 5% (*w*/*w*). The total flavonoid content of the samples was quantified using NaNO_2_-Al(NO_3_)_3_-NaOH spectrophotometry at 510 nm, with rutin as the standard, yielding a content of 62.0%.

UHPLC/Q-Orbitrap/HRMS analysis was conducted on an Ultimate 3000 UHPLC system coupled with a Q Exactive Plus HRMS (Thermo Fisher, Waltham, MA, USA). Detection and compound identification were conducted following previously described conditions [19].

### 2.3. Analysis of Blood-Entering Components

Test samples were obtained as previously described [20]. Ten male BALB/c mice (SPF-grade) were evenly allocated into two groups: a saline-treated control (*n* = 5) and an SSF-treated group (*n* = 5). The latter received SSF orally at 1.6 g/kg (10-fold the human clinical daily dose) for one week, while the control group was administered saline in equal volumes. On day 7, blood samples were collected via retro-orbital puncture 1 h post-administration, transferred to heparinized tubes, and gently inverted. Plasma was isolated by centrifugation at 3000 rpm for 10 min (4 °C) and preserved at −80 °C. To minimize inter-individual variability, plasma from each group was pooled before analysis. The absorbed constituents in plasma were identified by UHPLC/Q-Orbitrap/HRMS using the specified parameters.

### 2.4. Network Pharmacology Analysis

To identify potential targets of SSF for treating immunosuppression, the flavonoid components of SSF that enter the blood in their prototype form were first retrieved from the TCMSP database (https://www.tcmsp-e.com/tcmsp.php) and Swiss Target Prediction (http://swisstargetprediction.ch/) to obtain associated potential targets. Disease-related target genes were then searched using the keyword “Immunosuppression” in the GeneCards (https://www.genecards.org/), OMIM (https://omim.org/), and DisGeNET (https://disgenet.com/) databases. All targets were standardized using the UniProt database (https://www.uniprot.org/), and duplicates were removed. Venn diagrams were generated using the Jvenn online tool (https://jvenn.toulouse.inrae.fr/app/example.html, accessed on 30 July 2025) to visualize overlapping targets between SSF active components and immunosuppression-related genes. The intersection targets were processed in the STRING database for PPI analysis, visualized in Cytoscape (v3.10.0), and assessed using CytoNCA for network centrality metrics (BC, CC, DC). Core targets were screened based on median values to construct a subnetwork. The “Compound-Disease-Target” network was built in Cytoscape, integrating active SSF components and anti-immunosuppressive targets. GO functional annotation and KEGG pathway enrichment analysis were performed using the DAVID database (https://david.ncifcrf.gov/), with significance set at *p <* 0.05. Visualization was conducted through the Bioinformatics online platform (https://www.bioinformatics.com.cn/) and Chiplot (https://www.chiplot.online/).

### 2.5. NF-κB Luciferase Activity Assay

The THP1-Lucia NF-κB cell line stably integrates an NF-κB-regulated Lucia luciferase reporter, enabling the monitoring of NF-κB signaling pathway activation status [21]. WT and MyD88^CRISPR−/−^ THP1-Lucia NF-κB cells were provided by the School of Pharmaceutical Sciences, Tsinghua University (Beijing, China). For experiments, cells were plated in 96-well plates (1 × 10^5^ cells/well in 200 µL medium) and treated as follows: untreated control, LPS (100 ng/mL, positive control), or different concentrations of SSF or its monomeric compounds. After 18 h of incubation (37 °C, 5% CO_2_), NF-κB activation was measured by transferring 20 µL of supernatant to a white 96-well plate and mixing with 50 µL/well of coelenterazine-based detection reagent. Luminescent signals were recorded using a TECAN Infinite M Nano microplate reader (Tecan Group Ltd., Männedorf, Switzerland).

### 2.6. Experimental Design for Animal Studies

Sixty male BALB/c mice (8 weeks old, 20 ± 2 g) were obtained from SPF Biotechnology Co., Ltd. (Beijing, China; License No. SCXK 2024-0001) and maintained under controlled conditions (23 ± 2 °C, 55 ± 10% humidity, 12/12 h light/dark cycle) with ad libitum access to food and water. All procedures complied with the National Institutes of Health (NIH) Guidelines for Laboratory Animal Welfare and were approved by the Animal Ethics Committee of China Agricultural University (Approval No. AW91214202-2-03). After a one-week acclimatization, the mice were evenly divided into six groups (*n* = 10 each): blank (Con), model (CTX), positive control (LH), low (SSF-L), medium (SSF-M), and high (SSF-H)-dose SSF groups. The dosage of SSF was calculated based on the crude drug dose recommended by the Chinese Pharmacopoeia (2020) and a 5% flavonoid extraction rate, resulting in a human dose of 10.7–21.5 mg/kg. This corresponds to a mouse equivalent dose of 97.5–195 mg/kg. Experimental doses of 80–320 mg/kg were selected to assess dose-dependent therapeutic effect. The experimental procedures were as follows: the model and treatment groups were intraperitoneally injected with CTX (80 mg/kg) for the first three consecutive days to establish an immunosuppression model, while the blank group received saline. From day 4 to day 10, the positive control group was administered LH (40 mg/kg) via oral gavage once daily [22]. The three SSF-treated groups received oral doses of 80, 160, and 320 mg/kg SSF, respectively, whereas the blank and model groups were given an equivalent volume of saline. After seven days of treatment, blood samples were collected via retro-orbital bleeding under isoflurane anesthesia. The animals were then humanely euthanized by cervical dislocation, and the spleen, thymus, ileum, and cecal contents were harvested for analysis.

### 2.7. Body Weight and Immune Organ Indices

Body weight was recorded daily at a fixed time (10:00 ± 1 h). Upon completion of the experiment, the spleen and thymus were harvested, briefly washed in saline, blotted dry with filter paper, and weighed. Subsequently, the organs were photographed against a 1 cm × 1 cm grid background. Organ indices were derived using the following formula:

Organ index (mg/g) = Weight of spleen or thymus (mg)/Body weight (g)

### 2.8. Splenic Lymphocyte Proliferation Assay

Under sterile conditions, spleens were excised, washed twice in PBS, and gently homogenized using the plunger of a sterile syringe until no visible tissue fragments remained. The resulting cell suspension was filtered through a 200-mesh sieve and centrifuged at 1000 rpm for 5 min. After supernatant removal, erythrocytes were lysed by resuspending the pellet in RBC lysis buffer for 2–3 min, followed by neutralization with PBS and centrifugation. Splenocytes were suspended in RPMI-1640 medium at a density of 2 × 10^5^ cells/mL. Cells (100 µL/well) were plated in 96-well plates and incubated for 48 h with either ConA (5 µg/mL, T cell stimulant) or LPS (10 µg/mL, B-cell stimulant). Subsequently, lymphocyte proliferation was evaluated by adding CCK-8 solution and measuring absorbance at 450 nm.

### 2.9. Flow Cytometry

Staining involved spleen single-cell suspension with fluorescent antibodies (CD45-FITC, CD3-APC, CD4-PE, CD8-PerCP) for 30 min at 4 °C in the dark. After centrifugation (1000 rpm, 5 min), cells were washed with PBS and analyzed by flow cytometry (Cytek NL-CLC3000, Cytek Biosciences, Fremont, CA, USA) using SpectroFloCLC V1.0 software.

### 2.10. Detection of Serum Cytokines and Immunoglobulins

Mouse whole blood samples were left to clot at ambient temperature for 1 h, then centrifuged (3000 rpm, 15 min) to isolate the serum. Serum concentrations of IL-2, IL-6, IL-10, TNF-α, IFN-γ, IgM, and IgG were quantified via ELISA using standardized protocols from the kit manufacturer. For secretory IgA (sIgA) analysis in intestinal tissue, ileal segments were homogenized in saline solution (0.9% NaCl, 1:9 *w*/*v*) and centrifuged under identical conditions. The resultant 10% homogenate supernatant was collected for sIgA detection.

### 2.11. H&E and AB-PAS Staining

Spleen, thymus, and ileum samples were fixed in 4% PFA, paraffin-embedded, sectioned, and H&E-stained for histopathological analysis. Ileal morphology (villus height, crypt depth, VH/CD ratio) was analyzed using ImageJ (v1.53, NIH). Additionally, ileal sections underwent Alcian Blue-Periodic Acid-Schiff (AB-PAS) staining to assess mucus-secreting goblet cells. Whole-slide images were acquired using the Aperio Versa 8 research-grade digital slide scanner (Leica Biosystems, Wetzlar, Germany), and the positively stained area was quantified by calculating the mean integrated optical density (IOD) of AB-PAS reactivity.

### 2.12. Immunohistochemical (IHC) Analysis

Deparaffinized and rehydrated ileal sections underwent antigen retrieval (citrate buffer, pH 6.0, 25 min), peroxidase inactivation (3% H_2_O_2_, 20 min), and blocking (5% BSA, 30 min). Primary ZO-1 antibody (1:1000) was incubated overnight (4 °C). After washing, streptavidin-HRP (1:200, 50 min, RT) was applied. DAB (1 min) detected signals, followed by hematoxylin counterstaining for bright-field microscopy.

### 2.13. Western Blot Analysis

Proteins were extracted using RIPA buffer supplemented with protease and phosphatase inhibitors, followed by quantification via the BCA assay. The samples were then resolved on 12% SDS-PAGE gels and transferred to PVDF membranes. After blocking with 5% non-fat milk for 2 h, the membranes were incubated with primary antibodies overnight at 4 °C. Following TBST washes, the membranes were treated with HRP-conjugated secondary antibodies for 1 h. Protein signals were visualized using chemiluminescent detection (Cytiva ImageQuant 800, Amersham, Cytiva, Marlborough, MA, USA) and quantified with ImageJ.

### 2.14. 16S rRNA Sequencing

Microbial genomic DNA was extracted from cecal contents. The V3-V4 hypervariable region of the 16S rRNA gene was amplified using primers 338F and 806R. The purified amplicons were sequenced on an Illumina NextSeq 2000 (Illumina, Inc., San Diego, CA, USA), and subsequent bioinformatic processing and data visualization were conducted using the MajorBio Cloud platform (https://www.majorbio.com/).

### 2.15. Statistical Analysis

All data were analyzed with GraphPad Prism 8.0.2 and presented as mean ± standard deviation (SD). One-way ANOVA was applied for multi-group comparisons. Statistical significance was defined as *p* < 0.05, with indicators denoted as *p* < 0.05 (*), *p* < 0.01 (**), and *p* < 0.001 (***) or *p* < 0.05 (#), *p* < 0.01 (##), and *p* < 0.001 (###).

## 3. Results

### 3.1. Chemical Composition Analysis of SSF In Vitro and In Vivo

**Figure 1:**
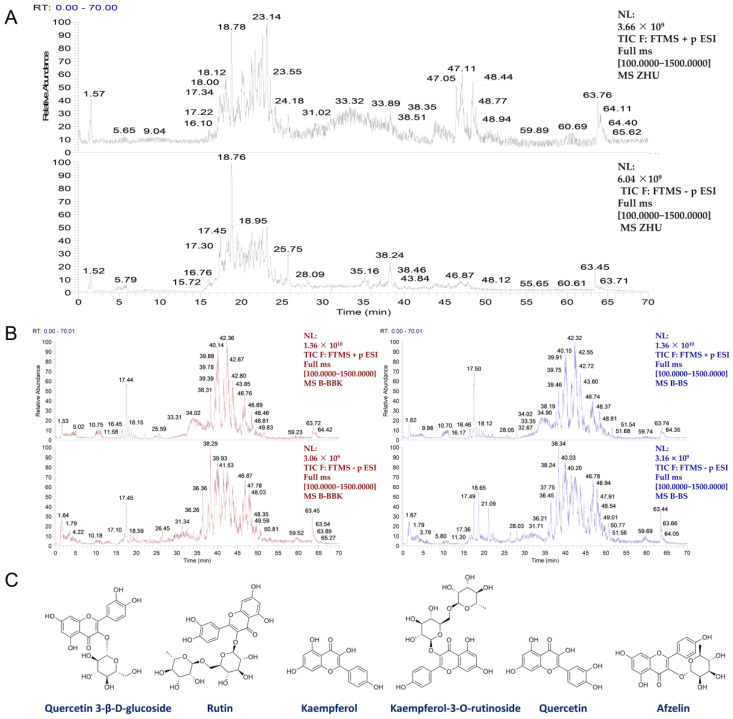
Characterization of chemical constituents of SSF in vitro and in vivo using UHPLC/Q-Orbitrap/HRMS. (**A**) Total ion chromatograms (TICs) of SSF in positive and negative ionization modes. (**B**) TICs of blank plasma (red) and SSF-containing plasma (blue) in both ionization modes. (**C**) Structures of prototype flavonoid components of SSF absorbed into the bloodstream.

The chemical constituents of SSF extracts were analyzed and identified using UHPLC/Q-Orbitrap/HRMS. Total ion chromatograms (TIC) of SSF were acquired in both positive and negative ion modes (Figure 1A). By integrating retention time (RT), MS/MS fragmentation patterns, and database comparisons, 119 chemical constituents were identified, including 20 flavonoid compounds that represented a significant proportion (Table 1). Detailed information on all compounds is provided in Supplementary Table S1. Of note, serum pharmacochemistry analysis detected 13 prototype SSF components in the blood after effectively eliminating interference from blank serum (Figure 1B, Supplementary Table S2). Most of these were flavonoids, featuring six representative compounds: quercetin-3β-D-glucoside, rutin, kaempferol, kaempferol-3-O-rutinoside, quercetin, and afzelin (molecular structures shown in Figure 1C).

### 3.2. SSF Attenuates CTX-Induced Weight Loss and Protects Immune Organ Damage in Mice

**Figure 2:**
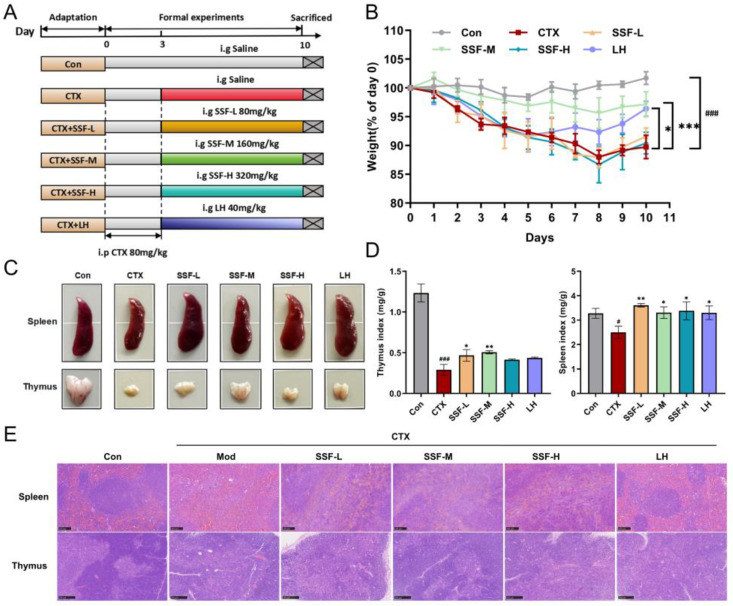
Effects of SSF on CTX-induced immune impairment in mice. (**A**) Schematic diagram of the in vivo experimental design. (**B**) Body weight change curve. (**C**) Representative macroscopic images of spleen and thymus. (**D**) Spleen and thymus indices. (**E**) Representative H&E-stained images of spleen and thymus (scale bars: spleen 100 μm; thymus 250 μm). Data are presented as mean ± SEM (*n* = 5). # *p* < 0.05, ### *p* < 0.001 vs. the Con group; * *p* < 0.05, ** *p* < 0.01, *** *p* < 0.001 vs. the Mod group.

The immunomodulatory effects of SSF were assessed using a CTX-induced immunosuppression mouse model (experimental design shown in Figure 2A). Compared to the Con group, CTX modeling significantly reduced body weight and markedly decreased spleen and thymus indices (Figure 2B–D). In contrast, the SSF-M and LH groups effectively reversed CTX-induced weight loss (Figure 2B). The spleen and thymus are vital immune organs rich in lymphocytes and macrophages, playing crucial roles in adaptive immunity (e.g., antibody production, T cell activation) and innate immune regulation. The administration of CTX caused the apparent atrophy of these organs, while SSF intervention significantly restored their morphology and organ indices (Figure 2C,D). Moreover, HE staining revealed that the Mod group exhibited reduced white pulp areas, blurred boundaries between red and white pulp, and sparse lymphocyte arrangement in the spleen; the thymus displayed indistinct cortico/medullary demarcation and decreased lymphocyte density (Figure 2E). In contrast, SSF treatment significantly improved the histological structure of the spleen and thymus, with pathological changes alleviated to nearly normal levels. These results demonstrate that SSF effectively protects immune organ function, thereby exerting immunomodulatory effects.

### 3.3. SSF Ameliorates CTX-Induced Immune Dysfunction

**Figure 3:**
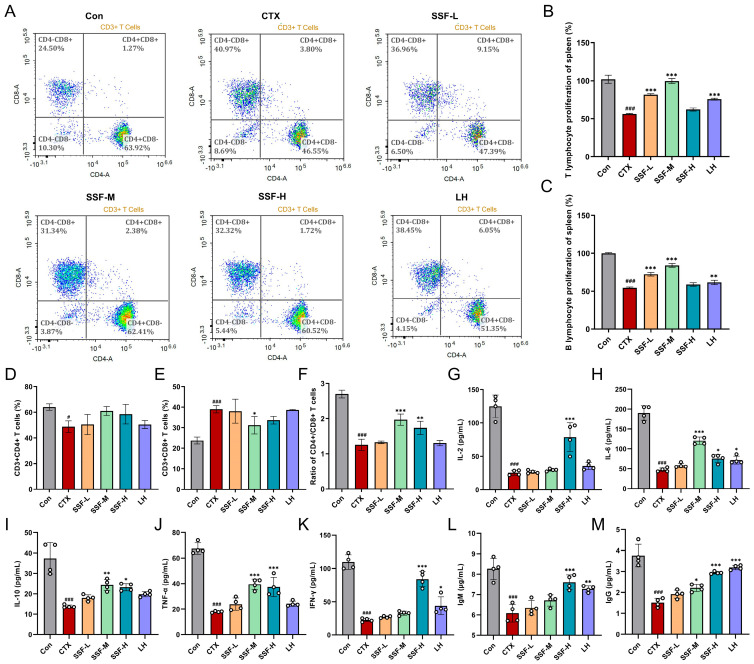
Effects of SSF on CTX-induced immune dysfunction in mice. (**A**) Splenic CD3^+^, CD4^+^, and CD8^+^ T cell subset proportions. (**B**) ConA-stimulated T cell proliferation. (**C**) LPS-stimulated B cell proliferation. (**D**) CD3^+^CD4^+^ and (**E**) CD3^+^CD8^+^ T cell percentages. (**F**) CD4^+^/CD8^+^ ratio. Serum levels of cytokines/immunoglobulins IL-2 (**G**), IL-6 (**H**), IL-10 (**I**), TNF-α (**J**), IFN-γ (**K**), IgM (**L**), and IgG (**M**). Data are presented as mean ± SEM (*n* = 4). # *p* < 0.05, ### *p* < 0.001 vs. the Con group; * *p* < 0.05, ** *p* < 0.01, *** *p* < 0.001 vs. the Mod group.

The proliferation and function of splenic lymphocytes serve as the most direct indicator of immune enhancement. Flow cytometry analysis of splenic lymphocyte subsets (Figure 3A) revealed that CTX treatment significantly altered the distribution characteristics of T lymphocyte subsets. CTX has been shown to markedly suppress the activity of T lymphocyte subsets. CD3^+^ T cells reflect global changes in T cell populations, while CD4^+^ and CD8^+^ T cells, as key functionally differentiated subsets, collaboratively regulate antigen-specific cellular immune responses. The CD4^+^/CD8^+^ ratio is a critical dynamic balance parameter for assessing immune activation or suppression status. Experimental data indicated that the Mod group exhibited a significant decrease in the proportion of CD4^+^ T lymphocytes and the CD4^+^/CD8^+^ ratio (Figure 3D,F). In contrast, the proportion of CD8^+^ T lymphocytes was markedly increased (Figure 3E). However, SSF intervention resulted in a non-significant upward trend in CD4^+^ T lymphocyte proportion. At the same time, the SSF-M and SSF-H groups significantly elevated the CD4^+^/CD8^+^ ratio, with the SSF-M group restoring the proportion of CD8^+^ T lymphocytes to normal levels (Figure 3D–F). Furthermore, in the ConA/LPS-stimulated lymphocyte proliferation assay, CTX significantly inhibited T and B lymphocyte proliferation compared to the Con group. In contrast, SSF-L and SSF-M groups exhibited significantly enhanced proliferative activity relative to the Mod group (Figure 3B,C). Additionally, cytokines, which are core immunotherapy targets, precisely regulate immune cell functions through multi-mechanistic interactions. SSF corrected the CTX-induced cytokine imbalance, significantly upregulating key cytokines such as IL-2, IL-6, IL-10, TNF-α, and IFN-γ (Figure 3G–K) while also reversing the reduced levels of IgM and IgG in CTX-treated mice (Figure 3L,M). These results suggest that SSF reverses CTX-mediated immunosuppression by synergistically regulating cellular immunity (T cell subset balance and lymphocyte proliferation) and humoral immunity (antibody and cytokine secretion).

### 3.4. SSF Restores Intestinal Barrier Integrity in CTX-Treated Mice

**Figure 4:**
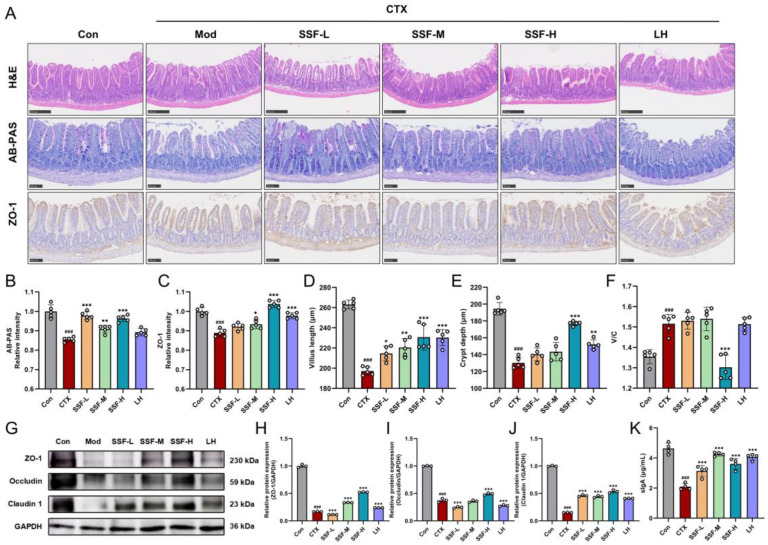
Effects of SSF on CTX-induced intestinal barrier integrity in mice. (**A**) Representative images of ileal H&E, AB-PAS staining, and ZO-1 immunohistochemistry (scale bars: H&E 250 μm; AB-PAS/ IHC 150 μm). (**B**) IOD value of AB-PAS-positive cells. (**C**) IOD value of ZO-1. Ileal villus height (**D**), crypt depth (**E**), and V/C ratio (**F**). (**G**) Immunoblotting of ZO-1, Occludin, and Claudin-1. Relative protein expression of ZO-1 (**H**), Occludin (**I**), and Claudin-1 (**J**). (**K**) Ileal sIgA content. Data are presented as mean ± SEM (*n* = 3). ### *p* < 0.001 vs. the Con group; * *p* < 0.05, ** *p* < 0.01, *** *p* < 0.001 vs. the Mod group.

Next, we assessed the protective effects of SSF on the intestinal barrier. Histopathological examination revealed intact and regularly arranged ileal villi with normal crypt morphology in the control group, whereas the model group displayed villus atrophy, structural loosening, and local disruption (Figure 4A). Quantitative analysis demonstrated significant reductions in villus height and crypt depth in the Mod group compared to the Con group. All SSF-treated and LH groups effectively restored these parameters to nearly normal levels but without significantly affecting the villus height/crypt depth (V/C) ratio (Figure 4D–F). Goblet cells and mucins visualized by AB-PAS staining showed significantly fewer positive signals in the model group, while SSF intervention increased AB-PAS-positive staining areas (Figure 4B). Immunohistochemistry and Western blot analysis further revealed that the expression levels of tight junction proteins (ZO-1, Occludin, and Claudin-1) were significantly downregulated in the Mod group compared to the Con group. SSF-M, SSF-H, and LH groups upregulated ZO-1 expression (Figure 4C). All SSF doses enhanced the expression of the three tight junction proteins in a dose-dependent manner (Figure 4G–J). Moreover, CTX modeling substantially suppressed the secretion of sIgA, a key mucosal immune effector molecule, while SSF administration restored sIgA levels (Figure 4K). These findings demonstrate that SSF improves intestinal structural integrity and enhances barrier function.

### 3.5. SSF Reshapes the Composition of Gut Microbiota

**Figure 5:**
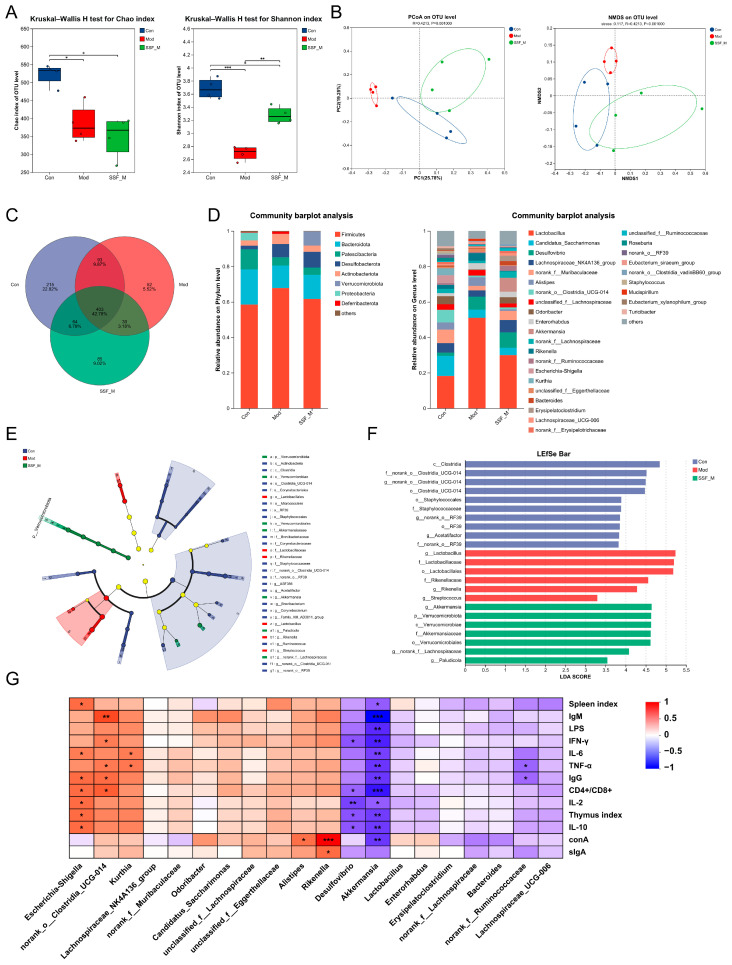
SSF reshapes gut microbiota dysbiosis to restore immune function in CTX-treated mice. (**A**) α-diversity assessed by Chao and Shannon indices (Kruskal–Wallis test). (**B**) β-diversity analyzed by PCoA and NMDS. (**C**) Venn diagram of OTU distribution. (**D**) Relative abundance of gut microbiota at phylum and genus levels. (**E**) LEfSe analysis identifying differentially abundant taxa. (**F**) LDA scores for significantly altered genera (LDA > 3). (**G**) Spearman correlation heatmap between 20 altered bacterial genera and immunosuppression-related parameters (blue: negative; red: positive). Data are presented as mean ± SEM (*n* = 4). * *p* < 0.05, ** *p* < 0.01, *** *p* < 0.001.

To clarify the reshaping characteristics of SSF on gut microbiota under immunosuppression, we investigated its regulatory effects on CTX-induced microbiota dysbiosis through 16S rRNA sequencing. The SSF-M group, showing significant efficacy, was selected for analysis based on pharmacodynamic evaluation. Alpha diversity analysis revealed that the Chao and Shannon indices in the Mod group were significantly reduced compared to the Con group, indicating CTX-induced depletion of microbial diversity. SSF-M intervention notably restored the Shannon index, although the Chao index showed no statistical difference (Figure 5A). Additionally, similar trends were observed for the Sobs, Ace, and Simpson indices (Supplementary Figure S2). Principal Coordinate Analysis (PCoA, PC1 = 25.78%, PC2 = 19.25%) and Non-Metric Multidimensional Scaling (NMDS) based on Bray–Curtis distances demonstrated significant separation among the three groups (Con, Mod, and SSF-M) in β-diversity (Figure 5B), highlighting structural differences in gut microbiota. The shared OTU distribution was visualized in the form of a Venn diagram (Figure 5C). *Firmicutes*, *Bacteroidota*, *Patescibacteria*, and *Desulfobacterota* were dominant at the phylum level. The Mod group exhibited increased relative abundances of *Firmicutes* and *Desulfobacterota*, while *Bacteroidota* and *Patescibacteria* were reduced. The SSF-M treatment significantly inhibited the overgrowth of *Firmicutes*. At the genus level, SSF-M upregulated beneficial commensals, such as the *Lachnospiraceae_NK4A136_group*, *norank_f__Muribaculaceae*, *Odoribacter*, and *Akkermansia*, while suppressing *Lactobacillus*, *Alistipes*, and *Rikenella*. Notably, the pathogenic genus *Desulfovibrio* was markedly enriched in the Mod group (Figure 5D). Using LEfSe analysis (LDA > 3), the signature taxa were identified as *c__Clostridia* in the Con group, *f__Lactobacillaceae* in the Mod group, and *f__Akkermansiaceae* in the SSF-M group (Figure 5E,F). A Spearman correlation analysis revealed associations between gut microbiota and immune indices. *Escherichia/Shigella*, *norank_o__Clostridia_UCG-014*, and *Kurthia* exhibited positive correlations with immune organ indices, immunoglobulins (IgM and IgG), and cytokines, while pathogenic bacteria like *Desulfovibrio* showed negative correlations (Figure 5G).

### 3.6. Network Pharmacology Analysis of SSF in Immunosuppression

**Figure 6:**
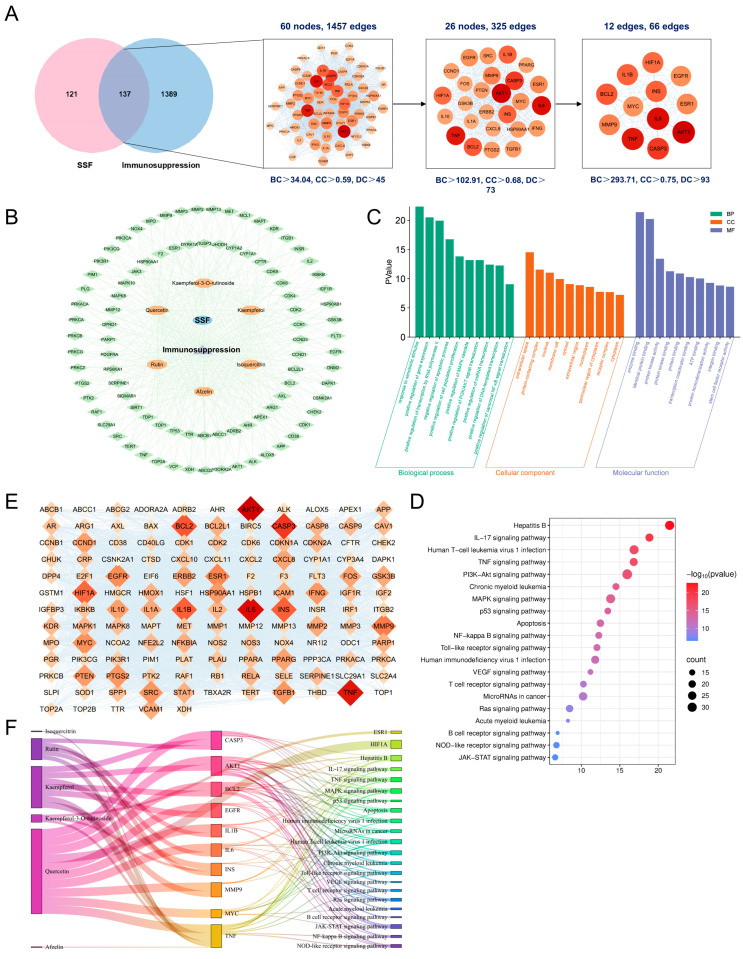
Network pharmacology analysis of SSF for treating immunosuppression. (**A**) Screening process of potential core targets of SSF against immunosuppression. (**B**) SSF component/target/immunosuppression network. Nodes represent the following: blue: SSF, orange: SSF components in serum; green: overlapping targets; purple: immunosuppression targets. (**C**) Histogram of GO annotation analysis. Bar length and color indicate the number of targets involved in related biological processes (BP: green; CC: orange; MF: purple). (**D**) Bubble plot of the top 20 enriched KEGG pathways. Color scale represents −log10 (*p*-value), and dot size indicates gene count per term. (**E**) PPI network visualization of 137 targets (node size reflects connectivity degree). (**F**) Component/target/pathway interaction network for SSF.

By integrating 258 potential target genes of SSF identified from the TCMSP and Swiss Target Prediction databases with 1526 immunosuppression-related targets from GeneCards, OMIM, and DisGeNET, a Venn analysis identified 137 overlapping targets (Figure 6A). The PPI network was constructed using the STRING platform, and through Cytoscape network topology analysis, 12 core targets (e.g., AKT1, IL6, TNF, CASP3, and BCL2) were screened (Figure 6A,E). Network visualization revealed that core targets exhibited higher connectivity and central distribution in the PPI network, suggesting their potential hub role in immune regulation. The constructed “component-target-disease” regulatory network shows that SSF exerts an immunomodulatory effect through multiple components and targets (Figure 6B). Gene ontology (GO) enrichment analysis indicated that the primary biological processes involved responses to external stimuli, positive regulation of gene expression, regulation of cell proliferation, and activation of the MAPK cascade. Cellular components were primarily enriched in the extracellular space and protein complexes, while molecular functions were predominantly associated with enzyme binding and kinase activity (Figure 6C). KEGG pathway analysis, focusing on the top 20 enriched pathways after excluding unrelated pathways, revealed significant enrichment in IL-17, TNF, PI3K-Akt, and NF-κB signaling pathways, which are considered the key pathways for SSF-regulating immunosuppression (Figure 6D). A Sankey diagram further illustrated the “component-target-pathway” relationships (Figure 6F). Given the central role of the NF-κB pathway in inflammatory immune responses, its validation was prioritized for subsequent investigation.

### 3.7. SSF Enhances Immune Response by Activating the TLR/MyD88/NF-κB Signaling Pathway

**Figure 7:**
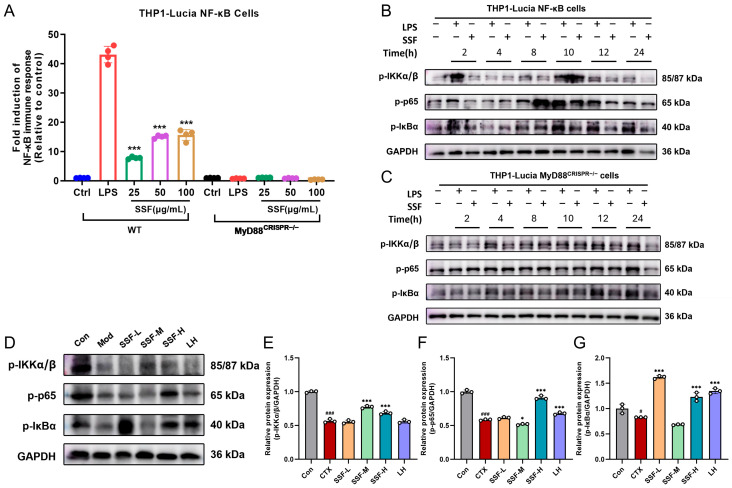
SSF activates the NF-κB signaling pathway to exert immunomodulatory effects. (**A**) NF-κB reporter gene activity in WT and MyD88^CRISPR−/−^ THP1-Lucia NF-κB cells stimulated with LPS (500 ng/mL) and SSF (50, 100, 200 μg/mL) for 18 h. Immunoblotting analysis of NF-κB pathway protein expression in WT (**B**) and MyD88^CRISPR−/−^ (**C**) THP1-Lucia NF-κB cells stimulated with LPS and SSF for 0–24 h. (**D**) Protein expression of p-IKKa/β, IκBα, and p-NF-κB p65 in tissues of SSF-treated mice. Relative quantification of p-IKKa/β/GAPDH (**E**), IκBα/GAPDH (**F**), and p-NF-κB p65/GAPDH (**G**). Data are presented as mean ± SEM (*n* = 3). # *p* < 0.05, ### *p* < 0.001 vs. the Con group; * *p* < 0.05, *** *p* < 0.001 vs. the LPS/Mod group.

To investigate the immunomodulatory mechanism of SSF, we first evaluated its activation effect on innate immune pathways using an NF-κB reporter gene system. The results demonstrated that SSF concentration significantly activated the NF-κB signaling pathway in THP1-Lucia NF-κB cells. At 25, 50, and 100 μg/mL concentrations, the activation increased to 7.8-fold, 15.1-fold, and 15.6-fold compared to the control group, respectively, showing a trend comparable to the positive control (500 ng/mL LPS). We further verified the upstream regulatory mechanisms using a MyD88-knockout cell model. Notably, the absence of MyD88 completely abolished the activation of the NF-κB pathway by both LPS and SSF (Figure 7A), confirming that SSF-mediated immunomodulation depends on the TLR/MyD88/NF-κB signaling axis. Western blot analysis with time-course experiments revealed that both LPS and SSF treatments rapidly induced the phosphorylation of key NF-κB pathway proteins (p-IKKa/β, IκBα, and p-NF-κB p65), peaking at 8–10 h post-treatment. This activation was entirely absent in MyD88-knockout cells (Figure 7B,C), further validating SSF’s reliance on the canonical TLR/MyD88/NF-κB pathway. Additionally, in a CTX-induced immunosuppression mice model, as expected, the expression levels of p-IKKa/β, IκBα, and p-NF-κB p65 in tissues of the Mod group were significantly reduced compared to the Con group. Intervention with SSF (all doses) and the LH group markedly reversed this inhibition (Figure 7D–G), indicating that SSF effectively reactivates the TLR/MyD88/NF-κB pathway to alleviate CTX-triggered immunosuppression and intestinal damage.

### 3.8. Screening and Evaluation of Potential Active Components in SSF

**Figure 8:**
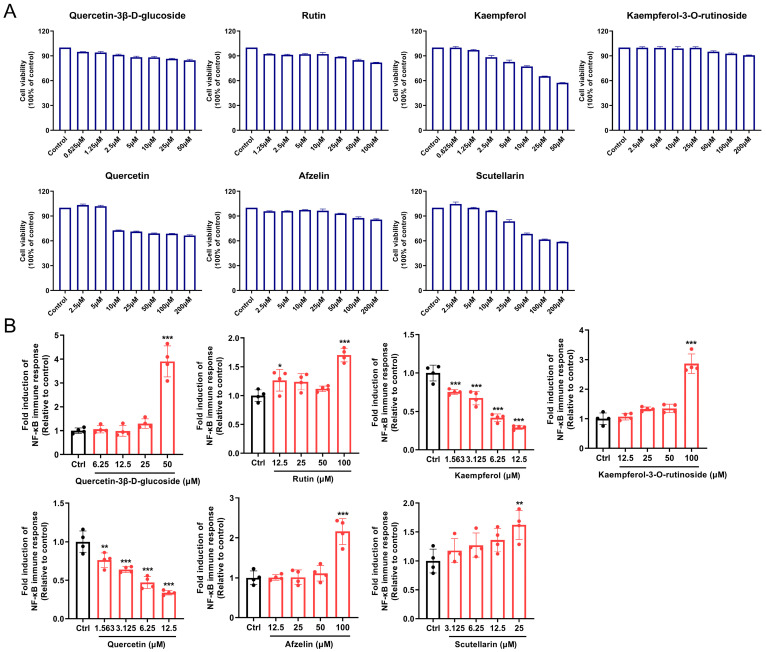
Activity evaluation of flavonoid components in SSF. (**A**) Viability of THP1-Lucia NF-κB cells co-incubated with seven compounds. (**B**) NF-κB reporter gene activity after 18 h treatment with the compounds. Data are presented as mean ± SEM (*n* = 4). * *p* < 0.05, ** *p* < 0.01, *** *p* < 0.001 vs. the Con group.

Based on serum pharmacochemistry analysis, six flavonoid prototypes and their primary metabolite scutellarin were identified in SSF. The effects of these components on NF-κB signaling pathway activation were evaluated using the THP1-Lucia NF-κB reporter system. Safe concentration ranges for each compound were preliminarily determined through CCK-8 assays. Following the determination of safe concentrations, luciferase activity measurements revealed that quercetin-3β-D-glucoside (isoquercitrin), rutin, kaempferol-3-O-rutinoside, afzelin, and scutellarin significantly activated NF-κB signaling at their highest tested concentrations. Notably, isoquercitrin demonstrated the strongest activation effect at 50 μM (Figure 8). These results indicate that flavonoids may serve as the critical active constituents underlying SSF’s immunomodulatory effects and its ability to improve intestinal mucosal damage.

## 4. Discussion

Immunosuppressive status significantly increases host susceptibility to pathogens, and rational immune stimulation strategies represent viable approaches for infection prevention [23]. Immunostimulatory therapy enhances the immune responses by promoting macrophage phagocytosis, dendritic cell antigen presentation, NK cell cytotoxicity, and T/B lymphocyte proliferation, while also stimulating the secretion of inflammatory cytokines and antibody production, thereby activating both innate and adaptive immunity [24]. Although *Senecio scandens*, as a traditional medicinal plant, has limited application due to the potential hepatotoxicity of its pyrrolizidine alkaloids (PAs), its flavonoid-rich fractions may have significant immunomodulatory potential, thereby circumventing the toxicity risks associated with alkaloids [25]. In fact, SSF demonstrated a favorable safety profile in both acute toxicity testing (LD50 > 5000 mg/kg) and a 90-day sub-chronic toxicity study in rats. Additionally, cytotoxicity assessments across multiple cell lines, including THP-1, RAW 264.7, HepG2, and HK-2, showed negligible impact on cell viability at concentrations ≤ 100 μg/mL, collectively confirming its safety advantages (unpublished data). The mechanisms of flavonoids as natural immunomodulators have been well validated [26,27,28]. For instance, flavonoids from *Astragalus complanatus* (FAC) not only activate macrophage phagocytosis but also significantly elevate the secretion levels of cytokines such as IL-1β, IL-6, and TNF-α via the NF-κB signaling pathway and induce iNOS expression to potentiate immune responses [29]. Similarly, *Sanghuangporus* flavonoids (PBF) promote the development of immune organs such as the thymus and spleen and synergistically strengthen cellular and humoral immune responses [30]. However, the immunomodulatory effects of SSF remain undetermined. Therefore, this study focuses on investigating the protective mechanisms of SSF against the CTX-induced immunosuppression.

### 4.1. SSF Alleviates CTX-Induced Systemic Immunosuppression

This study employed serum pharmacochemistry to identify the active components from SSF that were absorbed into the blood, including quercetin-3β-D-glucoside, rutin, kaempferol, kaempferol-3-O-rutinoside, quercetin, and afzelin. Existing studies have confirmed the immunomodulatory properties of rutin, kaempferol, and quercetin, supporting SSF’s role in immune enhancement [31,32,33,34]. The short-term administration of CTX rapidly induced immunosuppression in mice. The structural and functional integrity of immune organs serves as a primary indicator of systemic immune capacity [35]. In this study, histopathological analysis confirmed that CTX administration caused significant weight loss and atrophy of the thymus and spleen. Interestingly, the SSF intervention markedly alleviated immune organ damage and restored organ indices, consistent with prior reports [36]. Adaptive immunity relies on T and B lymphocytes [37]. CTX disrupts the Th1/Th2 balance, thereby impairing immune coordination and anti-infective capacity. Conversely, cytotoxic CD8^+^ T cells can eliminate virus-infected or tumor cells while contributing to immune regulation. CTX suppresses the CD4^+^/CD8^+^ T cell ratio to compromise immune surveillance. In addition, B cells mediate humoral immunity, wherein IgM serves as the frontline antibody in innate responses, while IgG acts as the primary effector in memory immunity, collectively sustaining long-term immune defense [38]. Our findings revealed that CTX significantly suppressed the proliferation of splenic T (CD4^+^, CD8^+^) and B lymphocytes, while also reducing immunoglobulin (IgG, IgM) concentrations, confirming its negative impact on both cellular and humoral immunity. Cytokine profiling showed decreased levels of IL-2, IL-6, IL-10, TNF-α, and IFN-γ, indicating broad-spectrum immune dysfunction. Collectively, we successfully established an immunosuppressed mouse model. Remarkably, SSF intervention improved most phenotypic and biochemical parameters in mice with CTX-induced immune impairment. These results align with previous reports by Zhang et al. and Li et al., further validating SSF’s potential to enhance immune function [39,40].

### 4.2. SSF Ameliorates CTX-Induced Intestinal Barrier Damage

The intestine serves not only as the primary site for nutrient absorption but also as the first-line immunological defense against pathogen invasion. Accumulating evidence highlights the critical association between intestinal barrier integrity and immune function [41]. In fact, CTX can disrupt the intestinal mucosal barrier, thinning the mucus layer, downregulating tight junction proteins, and reducing goblet cells and their secreted mucins, thereby exacerbating intestinal permeability and bacterial translocation risks. Our study showed that SSF markedly alleviated CTX-induced ileal villi atrophy, crypt structural damage, and inflammatory infiltration while significantly upregulating tight junction proteins (ZO-1, Occludin, and Claudin-1) to reinforce the physical barrier between intestinal epithelial cells. AB-PAS staining revealed that SSF increased goblet cell numbers and mucin coverage, thereby enhancing the protective capacity of the intestinal mucus layer. sIgA, an antibody secreted by mucosal epithelial cells, strengthens the mucosal barrier. The results indicated that SSF administration promoted sIgA secretion, thus improving immune barrier function [42]. These findings suggest that SSF restores intestinal barrier integrity by promoting immune homeostasis.

### 4.3. SSF Restores Gut Microbiota Homeostasis and Mediates Immune Regulation

The gut microbiota, known as the “second genome”, is pivotal in maintaining immune homeostasis through microbe/host interactions [43]. Immunosuppression induced by CTX can directly disrupt microbial balance, manifesting as reduced bacterial diversity and conditional pathogen overgrowth, which may further aggravate immune dysfunction [44]. On one hand, decreased microbial metabolites (e.g., SCFAs) impair energy supply for immune cells; on the other hand, the leakage of pathogen-derived components like lipopolysaccharide (LPS) into circulation may trigger chronic low-grade inflammation. Recent studies emphasize the profound influence of gut microbiota composition on immunotherapeutic efficacy, offering novel perspectives for mitigating immunosuppression through microbial modulation [45]. In this study, we assessed the regulatory effects of SSF on CTX-induced gut microbiota dysbiosis. The results showed that CTX treatment significantly reduced microbial diversity, as indicated by α-diversity indices, while SSF intervention partially restored microbial richness. Meanwhile, β-diversity analysis revealed distinct structural differences in the microbiota among treatment groups, confirming SSF’s ability to remodel gut microbiota. At the phylum level, CTX treatment significantly increased the *Firmicutes*/*Bacteroidetes* (F/B) ratio, in line with previous reports in *Lactobacillus acidophilus* LA85 and ginger polysaccharides [46,47]. Notably, SSF effectively reversed the F/B ratio imbalance. Elevated F/B ratios are generally linked to impaired intestinal barrier function, suggesting that SSF may enhance immunoregulatory functions by maintaining microbial homeostasis. At the genus level, SSF encouraged the proliferation of beneficial bacteria with immunomodulatory potential, such as the *Lachnospiraceae_NK4A136_group* and *Akkermansia*, while downregulating over-colonized genera like *Lactobacillus* and *Rikenella* to near-normal levels, thereby alleviating metabolic competition caused by excessive proliferation. Specifically, the *Lachnospiraceae_NK4A136_group* has been shown to improve intestinal barrier integrity and immune responses by stimulating short-chain fatty acid (SCFA) production, as exemplified by *Hericium erinaceus* polysaccharides alleviating CTX-induced immunosuppression through the upregulation of this genus [48]. Similarly, *Akkermansia muciniphila*, a well-recognized next-generation probiotic, can regulate mucus layer thickness, reinforce immune homeostasis, and mitigate metabolic disorders [49,50]. The enrichment of *Akkermansia* by SSF further supports its probiotic function. Furthermore, LEfSe analysis identified key differential microbiota across groups. Spearman correlation analysis revealed that *Escherichia*/*Shigella* (a pro-inflammatory genus) was positively correlated with most immune indices, possibly linked to enhanced host immune responses, while *norank_o__Clostridia_UCG-014* showed positive associations with serum immunoglobulin (IgM, IgG), IFN-γ, TNF-α, and CD4^+^/CD8^+^ ratios, suggesting its role in modulating T cell differentiation or antibody production. Recent reports have indicated that *norank_o__Clostridia_UCG-014* increases SCFA levels, thereby elevating the CD8^+^ T cell/Treg ratio and promoting TNF-α and IFN-γ secretion in the tumor microenvironment [51]. Conversely, *Desulfovibrio* abundance exhibited negative correlations with immune markers. As a harmful bacterium producing toxic hydrogen sulfide, it can inhibit mitochondrial respiration and induce intestinal epithelial apoptosis. Excessive *Desulfovibrio* may exacerbate gut inflammation and barrier dysfunction, undermining immune homeostasis [52]. Importantly, multiple studies have confirmed that reducing *Desulfovibrio* levels can ameliorate CTX-induced mucosal damage, such as the immunoprotective effects of yam protein through the suppression of this genus [53]. The similar regulatory trend of SSF further supports its microbiota-dependent effects in alleviating immunosuppression and intestinal damage.

### 4.4. SSF Activates the TLR/MyD88/NF-κB Signaling Pathway: Molecular Mechanisms

Network pharmacology has emerged as a powerful tool for elucidating drug mechanisms, particularly in the systematic study of bioactive compounds derived from natural medicines [54]. In this study, six blood-absorbable core flavonoids from SSF were subjected to network pharmacology analysis. We found that these active components are significantly associated with multiple critical immunoregulatory targets, including AKT1, IL6, TNF, CASP3, and BCL2. KEGG enrichment analysis further highlighted the NF-κB signaling pathway, which serves as a pivotal hub connecting innate and adaptive immunity and plays a central role by regulating inflammatory factor release, immune cell activation, and anti-infection responses [55,56]. The innate immune system detects pathogen-associated molecular patterns (PAMPs) via pattern recognition receptors (e.g., TLRs), initiating downstream cascades [57,58]. Experimental data demonstrated that SSF activates the TLR/MyD88-dependent pathway, facilitating MyD88/TLR binding and the subsequent phosphorylation of IKKα/β, which triggers IκBα degradation. This process releases p-NF-κB p65 for nuclear translocation, modulating the expression of cytokines (e.g., IL-6, TNF-α) and immune effectors. Consistent with this, SSF treatment upregulated p-IKKα/β, p-IκBα, and p-NF-κB p65 levels in mice. Taken together, these findings suggest that SSF counteracts the CTX-induced immunosuppressive state by activating the TLR/MyD88/NF-κB axis and ameliorates associated intestinal barrier damage.

Further in vitro screening of potential active components in SSF revealed that isoquercitrin exhibited the strongest activating effect on the NF-κB signaling pathway. This aligns with limited prior evidence indicating that it enhances humoral immunity by regulating B cell differentiation and antibody production [59]. This study further identified its involvement in activating the NF-κB signaling pathway. Despite these insights, investigations into the immunomodulatory functions of isoquercitrin remain relatively limited compared to other flavonoids, and existing experimental sample sizes need to be expanded to enhance the reliability of the conclusions. Subsequent research will employ an integrated approach leveraging molecular docking, CETSA, DARTS, and SPR technologies, combined with relevant gene-knockout mouse models, microbiota-depletion models, and fecal microbiota transplantation (FMT) models, to systematically elucidate the core mechanisms by which isoquercitrin mediates immunomodulatory and intestinal protective effects. This work will lay a theoretical foundation for developing targeted immunomodulators based on isoquercitrin and further drive in-depth exploration of its targeted delivery systems, bioactive compound isolation, novel formulation development, and clinical translation prospects.

## 5. Conclusions

In summary, this study comprehensively demonstrates that SSF alleviates CTX-induced immunosuppression and intestinal injury by activating the TLR/MyD88/NF-κB signaling axis and modulating the gut microbiota/immune system axis. Serum pharmacochemistry identified six key flavonoids that synergistically restore thymic and splenic function, improve T/B lymphocyte subset ratios, enhance immunoglobulin and cytokine secretion, repair intestinal barrier integrity, and promote beneficial bacterial colonization. Network pharmacology and experimental validation further revealed the mechanism by which SSF enhances immune responses, with isoquercitrin exhibiting the strongest NF-κB-activating potential. These findings lay a scientific foundation for the development of SSF-derived immune enhancers, dietary supplements, or feed additives, with potential applications in post-chemotherapy immune reconstitution and livestock health management. Future research should focus on developing targeted delivery systems for isoquercitrin (e.g., nanocarrier modification) and exploring synergistic interactions between SSF and other immunomodulators, particularly in the context of tumor immunotherapy. These investigations will facilitate the translation of SSF into precision immunomodulatory therapeutics.

## Figures and Tables

**Table 1:** The principal component of *Senecio scandens* flavonoids.

Number	Name	Formula	Annot. Delta Mass [ppm]	Calc. MW	*m*/*z*	RT [min]	Reference Ion
1	Quercetin	C15 H10 O7	0.19	302.04271	303.04999	21.644	[M+H]+1
2	Isoquercitrin	C21 H20 O12	0.4	464.09566	463.0882	21.645	[M−H]-1
3	Rutin	C27 H30 O16	−0.02	610.15337	609.14587	21.298	[M−H]-1
4	Kaempferol	C15 H10 O6	0.66	286.04793	287.05521	23.558	[M+H]+1
5	Astragalin	C21 H20 O11	0.09	448.1006	447.09308	22.548	[M−H]-1
6	Afzelin	C21 H20 O10	−0.15	432.10558	431.09818	23.559	[M−H]-1
7	Kaempferol-3-O-rutinoside	C27 H30 O15	0.58	594.15882	593.15131	22.051	[M−H]-1
8	Kaempferitrin	C27 H30 O14	−0.05	578.16353	577.15625	21.841	[M−H]-1
9	5,7-dihydroxy-2-phenyl-4H-chromen-4-one	C15 H10 O4	0.48	254.05803	255.06531	22.626	[M+H]+1
10	Quercetin-3-O-beta-glucopyranosyl-6′-acetate	C23 H22 O13	0.28	506.10618	505.09891	22.114	[M−H]-1
11	Apigenin 7-O-glucuronide	C21 H18 O11	1.31	446.0855	447.09293	22.816	[M+H]+1
12	Vicenin III	C26 H28 O14	0.77	564.14834	565.15588	20.303	[M+H]+1
13	Daidzein	C15 H10 O4	0.55	254.05805	255.06531	23.127	[M+H]+1
14	Engeletin	C21 H22 O10	−0.37	434.12114	433.11386	22.862	[M−H]-1
15	Eriodictyol	C15 H12 O6	0.25	288.06346	287.05606	24.94	[M−H]-1
16	Luteolin	C15 H10 O6	−0.41	286.04762	285.04034	25.48	[M−H]-1
17	Astilbin	C21 H22 O11	0.37	450.11638	449.1091	21.898	[M−H]-1
18	3,5,7-trihydroxy-2-phenyl-4H-chromen-4-one	C15 H10 O5	0.06	270.05284	271.06018	27.284	[M+H]+1
19	Naringenin	C15 H12 O5	−0.12	272.06844	271.0611	26.919	[M−H]-1
20	Didymin	C28 H34 O14	0.44	594.19512	595.20239	20.432	[M+H]+1

## Data Availability

The data are available in the article itself and its Supplementary Materials.

## Supplementary Materials

The following supporting information can be downloaded at https://www.mdpi.com/article/10.3390/nu17152540/s1: Table S1: The detailed information on 119 components of SSF. Table S2: The detailed information on 13 flavonoid components in SSF that enter the blood as prototypes. Figure S1: Schematic overview of the experimental workflow. Figure S2: Changes in gut microbiota of immunocompromised mice with SSF treatment. Figure S3: Immunoblotting raw images.
