# Supplementary material for: Flavonoid Extract of Senecio scandens Buch.-Ham. Ameliorates CTX-Induced Immunosuppression and Intestinal Damage via Activating the MyD88-Mediated Nuclear Factor-κB Signaling Pathway
Source: Nutrients. 2025 Aug 1;17(15):2540. doi: 10.3390/nu17152540 (PMC12348433; doi:10.3390/nu17152540)
Supplement: Supplementary file 1 [file nutrients-17-02540-s001.zip › nutrients-3730665-supplementary.pdf]

Flavonoid Extract of *Senecio Scandens* Buch.-Ham. Ameliorates CTX-Induced Immunosuppression and Intestinal Damage via Activating the MyD88-Mediated Nuclear Factor-κB Signaling Pathway

Xiaolin Zhu [1,2,3], Lulu Zhang [1,2,3], Xuan Ni [1,2,3], Jian Guo [1,2,3], Yizhuo Fang [1,2,3], Jianghan Xu [1,2,3], Zhuo Chen [1,2,3,*] and Zhihui Hao [1,2,3,*]

[1] Innovation Centre of Chinese veterinary medicine, College of Veterinary Medicine, China Agricultural University, Beijing 100193, China

[2] State Key Laboratory of Veterinary Public Health and Safety, College of Veterinary Medicine, China Agricultural University, Beijing 100193, China

[3] Key Biology Laboratory of Chinese Veterinary Medicine, Ministry of Agriculture and Rural Affairs, Beijing 100193, China

E-mail addresses: zhuxiaolin1103@163.com (X.Z.); zhanglulu@cau.edu.cn (L.Z.); nixuandream@outlook.com (X.N.); guojiancloud@sina.com (J.G.); fyz980088310@gmail.com (Y.F.); 15750510829@163.com (J.X.)

*Corresponding authors: chenzhuo0523@yeah.net (Z.C.); haozhihui@cau.edu.cn (Z.H.)

Supplementary Data

Table S1. The detailed information on 119 components of SSF.

Number	Name	Formula	Annot. DeltaMass [ppm]	Calc. MW	m/z	RT [min]	Reference Ion	Fragment Ions (MS/MS)
1	Chlorogenic acid	C16 H18 O9	-0.07	354.09506	707.18298	18.795	[2M-H]-1	191.05621 179.03520 173.04550 135.04512 93.03440
2	Ethyl caffeate	C11 H12 O4	-0.88	208.07337	207.06606	25.738	[M-H]-1	207.06624 179.03502 161.02443 137.02429 135.04518
3	Quercetin	C15 H10 O7	0.19	302.04271	303.04999	21.644	[M+H]+1	303.0499 257.04434 229.04955 153.01830 137.02347
4	D-(-)-Quinic acid	C7 H12 O6	-0.82	192.06323	191.05595	18.787	[M-H]-1	191.05615 127.04002 93.03444 87.00860 85.02934
5	Isoquercitrin	C21 H20 O12	0.4	464.09566	463.0882	21.645	[M-H]-1	303.05002 285.04013 257.04404 229.04938 153.01820
6	Rutin	C27 H30 O16	-0.02	610.15337	609.14587	21.298	[M-H]-1	300.02753 271.02481 255.02988 151.00348 178.99879
7	3,5-Dicaffeoylquinic acid	C25 H24 O12	-0.24	516.12665	515.11932	22.429	[M-H]-1	191.05615 179.03502 173.04520 161.02440 135.04510
8	Phenylglyoxylic acid	C8 H6 O3	-0.93	150.03155	149.0242	18.437	[M-H]-1	149.02434 121.02942 95.05013 93.03425 77.03961
9	Kaempferol	C15 H10 O6	0.66	286.04793	287.05521	23.558	[M+H]+1	287.05505 213.05426 165.01834 153.01834 121.02855
10	Astragalin	C21 H20 O11	0.09	448.1006	447.09308	22.548	[M-H]-1	447.09344 300.02753 284.03253 255.02986 227.03467
11	Afzelin	C21 H20 O10	-0.15	432.10558	431.09818	23.559	[M-H]-1	431.09818 284.03275 255.03001 227.03505 185.06137
12	Asarylaldehyde	C10 H12 O4	1.43	196.07384	197.08112	20.167	[M+H]+1	197.08109 169.04985 151.07486 123.04417 111.04442
13	Kaempferol-3-O-rutinoside	C27 H30 O15	0.58	594.15882	593.15131	22.051	[M-H]-1	593.15302 285.04022 255.02998 227.03505 151.00343

14	6-Methoxysalicylic acid	C8 H8 O4	-0.94	168.0421	167.03482	5.749	[M-H]-1	123.04502 109.02495 108.02158 95.05050 68.99565
15	p-Hydroxybenzaldehyde	C7 H6 O2	1.47	122.03696	123.04424	20.168	[M+H]+1	123.04420 105.03413 95.04936 67.05458 65.03896
16	Corchorifatty acid F	C18 H32 O5	-0.42	328.22484	327.21756	26.886	[M-H]-1	309.20786 239.12868 211.1340 171.10263 137.09708
17	Caffeic acid	C9 H8 O4	-0.5	180.04217	179.03487	19.431	[M-H]-1	135.04512 134.03725 133.02960 117.03449 107.05011
18	Coumarin	C9 H6 O2	0.63	146.03687	147.04417	21.888	[M+H]+1	147.04411 120.05259 119.04923 91.05436 65.03892
19	Cryptochlorogenic acid	C16 H18 O9	-0.45	354.09492	353.08765	18.306	[M-H]-1	191.05618 179.03502 173.04547 135.04515 93.03447
20	Homogentisic acid	C8 H8 O4	-0.96	168.0421	167.03482	18.109	[M-H]-1	123.04510 149.02385 95.05020 77.03959 121.02931
21	Protocatechualdehyde	C7 H6 O3	-1.24	138.03152	137.02425	17.598	[M-H]-1	137.02435 108.02168 119.01376 136.01648 81.03447
22	3-Methylcatechol	C7 H8 O2	-1.31	124.05227	123.04499	5.732	[M-H]-1	122.03746 121.02947 108.02158 95.01361 93.03442
23	Benzoic acid	C7 H6 O2	-0.92	122.03667	121.02931	19.467	[M-H]-1	122.03262 121.0294 108.02161 93.03445 70.75092
24	4-Hydroxybenzaldehyde	C7 H6 O2	1.47	122.03696	123.04424	18.433	[M+H]+1	95.04932 105.04462 77.03880 65.03542 123.04417
25	Azelaic acid	C9 H16 O4	-0.58	188.10475	187.09747	22.886	[M-H]-1	169.08702 143.10745 125.09705 97.06576 83.05011
26	Riboflavin	C17 H20 N4 O6	1.12	376.1387	377.146	19.644	[M+H]+1	359.13419 243.08789 198.06654 216.07703 172.08705
27	trans-3-Indoleacrylic acid	C11 H9 N O2	0.88	187.06349	188.07077	17.572	[M+H]+1	144.08102 146.06018 117.06999 170.06020 118.06525
28	4-Coumaric acid	C9 H8 O3	1.15	164.04753	165.05481	21.887	[M+H]+1	147.04414 119.04926 91.05439 117.03374 164.04276

29	Esculetin	C9 H6 O4	-0.1	178.02659	177.01921	19.276	[M-H]-1	149.06055 105.03441 121.02951 133.02946 89.03956
30	5-Hydroxymethyl -2-furaldehyde	C6 H6 O3	0.93	126.03181	127.03907	18.151	[M+H]+1	109.02861 81.03368 53.03912 97.02859 71.04935
31	Skimmin	C15 H16 O8	0.01	324.08452	325.0918	20.862	[M+H]+1	307.0813 163.07500 145.02852 117.03353 135.04411
32	L-Tryptophan	C11 H12 N2 O2	0.89	204.09006	205.09734	17.574	[M+H]+1	188.07079 170.06029 146.06017 144.08095 118.06519
33	1-Linoleoyl glycerol	C21 H38 O4	0.39	354.27715	355.28442	45.59	[M+H]+1	337.27396 263.23718 245.22652 109.10134 81.07011
34	1-Palmitoylglycer ol	C19 H38 O4	0.32	330.27712	331.28439	38.234	[M+H]+1	313.27371 239.23782 95.08568 57.07039 71.08580
35	Butyl benzoate	C11 H14 O2	0.96	178.09955	179.10683	22.254	[M+H]+1	105.07006 123.04439 161.09615 133.10133 107.04932
36	Galactose	C6 H12 O6	-0.7	180.06326	179.05598	18.439	[M-H]-1	89.02431 101.02419 71.01369 119.05013 135.04515
37	Adenine	C5 H5 N5	2	135.05477	136.06204	1.528	[M+H]+1	119.03535 110.06033 81.07014 57.07024 94.06532
38	4,5-Dicaffeoylqui nic acid	C25 H24 O12	1.03	516.12731	517.13458	22.846	[M+H]+1	163.03908 135.04411 117.03358 160.11214 319.08218
39	Choline	C5 H13 N O	1.81	103.0999	104.10718	1.499	[M+H]+1	104.10709 87.06821 60.08121 58.06558 69.03378
40	Adenosine	C10 H13 N5 O4	1.29	267.0971	268.10437	1.546	[M+H]+1	136.06194 119.03540 85.02853 55.01821 57.03397
41	2,3,4,9-Tetrahydr o-1H-β-carboline-3-carboxylic acid	C12 H12 N2 O2	0.77	216.09004	217.09732	18.914	[M+H]+1	156.08112 144.08101 130.06537 117.07006 145.08444
42	3-oxoindane-1-ca rboxylic acid	C10 H8 O3	0.55	176.04744	177.05489	20.419	[M+H]+1	149.05989 123.04420 145.02858 117.03362 89.03872
43	Shogaol	C17 H24 O3	0.73	276.17275	277.18002	31.887	[M+H]+1	137.05989 161.09534 93.07012 81.07011 231.17444

44	α,α-Trehalose	C12 H22 O11	-0.14	342.11616	341.10886	1.523	[M-H]-1	179.05614 161.04619 143.03543 119.03487 101.02433
45	Indole	C8 H7 N	0.9	117.05795	118.06523	17.573	[M+H]+1	91.05443 77.03876 65.03889 95.04840 59.07346
46	9-Oxo-10(E),12(E)-octadecadienoic acid	C18 H30 O3	0.74	294.21971	295.22699	28.09	[M+H]+1	277.21631 185.13408 123.08070 249.22118 165.12753
47	3-Hydroxybenzoic acid	C7 H6 O3	-1.24	138.03152	137.02425	23.034	[M-H]-1	93.03439 109.02042 65.03951 94.03778 137.02428
48	L-Phenylalanine	C9 H11 N O2	2.05	165.07932	166.08659	10.966	[M+H]+1	149.05933 131.04938 120.08089 103.05434 93.07006
49	4-(3,4-Dihydroxyphenyl)-6,7-dihydroxy-2-naphthoic acid	C17 H12 O6	-0.21	312.06332	311.05603	26.334	[M-H]-1	267.06616 250.08320 211.07602 193.06541 133.02943
50	Kaempferitrin	C27 H30 O14	-0.05	578.16353	577.15625	21.841	[M-H]-1	431.09772 285.04050 257.04514 213.05559 191.05597
51	12-oxo Phytodienoic Acid	C18 H28 O3	-0.11	292.20381	293.21109	26.887	[M+H]+1	275.20002 257.19043 163.11127 119.08568 107.08568
52	Linolenic acid ethyl ester	C20 H34 O2	0.78	306.25612	307.2634	46.005	[M+H]+1	261.22095 243.21088 121.10136 67.05457 81.07010
53	(-)-Caryophyllene oxide	C15 H24 O	0.88	220.18291	221.19019	29.202	[M+H]+1	203.17957 161.13278 147.11691 109.10131 105.07003
54	Ethyl benzoate	C9 H10 O2	0.86	150.06821	151.07549	20.171	[M+H]+1	123.04423 105.04495 95.04935 77.03870 65.03903
55	Sucrose	C12 H22 O11	-0.23	342.11613	341.10886	16.058	[M-H]-1	161.02457 191.05615 119.03485 89.02432 101.02428
56	3',4'-Dihydroxyphenylacetone	C9 H10 O3	-1.06	166.06282	165.05554	18.07	[M-H]-1	121.02931 109.02401 149.02385 123.04510 125.06016

57	Shikimic acid	C7 H10 O5	-2.01	174.05247	173.0452	18.885	[M-H]-1	155.03487 137.02434 71.01371 111.04497 93.03442
58	5,7-dihydroxy-2-phenyl-4H-chromen-4-one	C15 H10 O4	0.48	254.05803	255.06531	22.626	[M+H]+1	211.07529 227.05987 153.07011 199.07549 213.05460
59	4-Hydroxyphenyl acetic acid	C8 H8 O3	0.94	152.04749	153.05476	19.783	[M+H]+1	151.03932 135.04414 111.04414 79.05439 83.04926
60	D-(-)-Fructose	C6 H12 O6	-0.7	180.06326	179.05598	1.487	[M-H]-1	131.03471 119.03484 101.02432 89.02437 152.91759
61	Quercetin-3-O-beta-glucopyranosyl-6'-acetate	C23 H22 O13	0.28	506.10618	505.09891	22.114	[M-H]-1	463.0863 301.03403 271.02496 255.02992 151.00375
62	Apigenin 7-O-glucuronide	C21 H18 O11	1.31	446.0855	447.09293	22.816	[M+H]+1	271.06024 243.06506 153.01840 119.04926 107.04925
63	Orsellinic acid	C8 H8 O4	0.7	168.04238	169.04965	18.427	[M+H]+1	151.03915 123.04418 107.08566 95.04935 67.05456
64	Phytosphingosine	C18 H39 N O3	0.84	317.29326	318.30054	39.545	[M+H]+1	282.27921 300.28998 264.26938 95.08576 60.04483
65	Isoferulic acid	C10 H10 O4	-0.8	194.05775	193.05048	26.287	[M-H]-1	178.02678 121.02938 111.00878 93.03440 108.02158
66	(2E)-3-(3,4-dimethoxyphenyl)prop-2-enoic acid	C11 H12 O4	0.91	208.07375	209.08102	23.011	[M+H]+1	191.07037 177.05479 149.09621 121.06493 103.05436
67	2,3-Dihydroxybenzoic acid	C7 H6 O4	-0.89	154.02647	153.0192	15.619	[M-H]-1	109.02935 108.02159 81.03449 91.01874 65.03970
68	2'-O-Methyladenosine	C11 H15 N5 O4	1.34	281.11278	282.12006	15.965	[M+H]+1	150.07755 136.06197 119.03522 101.05981 69.03379
69	Esculin	C15 H16 O9	-0.58	340.07924	339.07187	18.042	[M-H]-1	177.01935 161.02446 133.02960 105.03456 179.03572

70	Vicenin III	C26 H28 O14	0.77	564.14834	565.15588	20.303	[M+H]+1	529.13489 511.12320 379.08148 427.10254 325.07074
71	Isophthalic acid	C8 H6 O4	-1.46	166.02637	165.01909	17.316	[M-H]-1	121.02942 93.03443 108.02161 94.03781 68.99563
72	2-Isopropylmalic acid	C7 H12 O5	-1.35	176.06824	175.06096	17.886	[M-H]-1	157.05054 131.07133 113.06071 115.03994 85.06576
73	3,4,5-trihydroxycyclohex-1-ene-1-carboxylic acid	C7 H10 O5	-1.49	174.05256	173.04529	16.247	[M-H]-1	155.03503 137.02434 111.08134 93.03442 71.01366
74	3-(4-Hydroxy-5-oxo-3-phenyl-2,5-dihydro-2-furanyl) propanoic acid	C13 H12 O5	0.59	248.06862	249.0759	18.43	[M+H]+1	231.06506 203.07019 161.05998 133.06503 123.04420
75	Acetophenone	C8 H8 O	1.38	120.05768	121.06496	19.649	[M+H]+1	103.0543 95.04926 91.05437 105.04482 77.03873
76	Daidzein	C15 H10 O4	0.55	254.05805	255.06531	23.127	[M+H]+1	227.07019 213.05472 199.07536 153.07008 91.05444
77	Kynurenic acid	C10 H7 N O3	0.87	189.04276	190.05003	18.218	[M+H]+1	162.05499 146.06003 144.04448 89.03839 116.04948
78	Fraxin	C16 H18 O10	-0.54	370.0898	369.08252	19.282	[M-H]-1	207.05093 193.05090 191.05563 129.01929 137.02446
79	8-Hydroxyquinoline	C9 H7 N O	0.65	145.05286	146.06013	22.604	[M+H]+1	129.06979 119.04928 118.06522 91.05439 105.07018
80	Stachyose	C24 H42 O21	0.49	666.22218	665.2149	1.523	[M-H]-1	323.09818 101.02433 113.02435 179.05617 89.0243
81	Engeletin	C21 H22 O10	-0.37	434.12114	433.11386	22.862	[M-H]-1	269.04562 287.05591 178.99861 152.01151 151.00357
82	Linoleoyl Ethanolamide	C20 H37 N O2	0.18	323.28249	324.28976	43.822	[M+H]+1	306.27809 245.22775 263.23788 62.08044 81.07007
83	4-Methylumbelliferone	C10 H8 O3	-1.2	176.04713	175.03986	22.194	[M-H]-1	158.03743 147.04515 119.05005 129.03456 101.03939

84	L-(-)-Malic acid	C4 H6 O5	-0.53	134.02145	133.01418	1.518	[M-H]-1	115.00356 107.03623 89.02435 71.01371 72.99296
85	3-Methoxybenzaldehyde	C8 H8 O2	0.73	136.05253	137.0598	24.458	[M+H]+1	122.07269 109.10145 94.05439 69.03378 79.05442
86	2-Pyrrolidinecarboxylic acid	C5 H9 N O2	1.77	115.06353	116.07081	1.536	[M+H]+1	98.07151 85.02872 72.04403 70.06543 55.05473
87	Emodin	C15 H10 O5	-0.28	270.05275	269.04547	26.33	[M-H]-1	241.05077 225.05562 197.06091 181.15991 169.06577
88	Catechol	C6 H6 O2	-2.01	110.03656	109.02928	15.598	[M-H]-1	108.02158 91.01886 93.29037 81.03443 65.03956
89	Resorcinol	C6 H6 O2	-1.9	110.03657	109.02928	18.089	[M-H]-1	108.02159 77.88170 109.03002 81.03455 107.05008
90	Eriodictyol	C15 H12 O6	0.25	288.06346	287.05606	24.94	[M-H]-1	269.04547 241.14484 151.00359 135.04512 107.01375
91	Trigonelline HCl	C7 H7 N O2	1.83	137.04793	138.05521	1.519	[M+H]+1	120.03864 92.04952 95.08570 65.03889 79.05434
92	Pinolenic acid	C18 H30 O2	-1.38	278.2242	277.21692	38.429	[M-H]-1	259.20703 127.0781 71.01340 69.29658 59.01371
93	Dodecanedioic acid	C12 H22 O4	-1	230.15158	229.1443	29.047	[M-H]-1	211.13399 185.06081 167.14409 139.11279 156.05783
94	Guanine	C5 H5 N5 O	1.87	151.04969	152.05699	2.86	[M+H]+1	123.04422 135.03030 95.08566 81.07014 69.07020
95	Palmitoyl ethanolamide	C18 H37 N O2	0.48	299.28257	300.28985	45.182	[M+H]+1	282.27948 239.23613 159.11690 175.14798 62.06044
96	Coniferylaldehyde	C10 H10 O3	1.43	178.06325	179.07053	22.984	[M+H]+1	161.05978 147.04414 133.06490 119.04927 91.05438
97	Luteolin	C15 H10 O6	-0.41	286.04762	285.04034	25.48	[M-H]-1	241.05118 217.05070 199.04012 175.04002 151.00357
98	Astilbin	C21 H22 O11	0.37	450.11638	449.1091	21.898	[M-H]-1	315.07159 285.04028 178.99881 152.01151 151.00356
99	1,3-Dicaffeoylquinic acid	C25 H24 O12	1.62	516.12761	517.13489	18.819	[M+H]+1	325.07056 283.06021 255.06505 163.03908 135.04414
100	Methyl	C11 H12 O4	0.91	208.07375	209.08102	16.269	[M+H]+1	177.05479 149.05992 121.06498 131.04935 91.05441

	4-hydroxy-3-methoxycinnamate							
101	α-Linolenoyl ethanolamide	C20 H35 N O2	0.64	321.26698	322.27426	41.333	[M+H]+1	243.21124 261.22165 81.07010 95.08569 62.06048
102	3,5,7-trihydroxy-2-phenyl-4H-chromen-4-one	C15 H10 O5	0.06	270.05284	271.06018	27.284	[M+H]+1	189.11209 217.14291 153.01834 119.04937 107.08569
103	Sedanolide	C12 H18 O2	1.17	194.13091	195.13818	26.925	[M+H]+1	177.12772 149.13266 121.06508 105.07002 91.05434
104	Pantothenic acid	C9 H17 N O5	0.95	219.11088	220.11826	16.278	[M+H]+1	202.10736 184.09714 176.10707 124.07589 90.05516
105	Indole-3-acrylic acid	C11 H9 N O2	0.88	187.06349	188.07077	16.717	[M+H]+1	170.05997 144.08133 143.07317 109.02872 91.05442
106	Oleoyl ethanolamide	C20 H39 N O2	0.67	325.2983	326.30557	45.877	[M+H]+1	308.29572 284.29504 95.08571 109.10123 62.06044
107	Monoolein	C21 H40 O4	-0.11	356.29262	357.2999	47.149	[M+H]+1	339.28928 247.24196 265.25290 135.11673 121.10127
108	Nicotinic acid	C6 H5 N O2	1.69	123.03224	124.03951	2.588	[M+H]+1	123.05544 124.03947 96.04450 80.04964 78.03398
109	Methyl cinnamate	C10 H10 O2	0.7	162.06819	163.07547	21.119	[M+H]+1	147.04419 131.04938 117.03363 103.05436 91.05441
110	Linoleic acid	C18 H32 O2	-1.68	280.23976	279.23248	40.676	[M-H]-1	279.23303 261.22293 96.95981 78.95885 71.05367
111	4-Ethylbenzaldehyde	C9 H10 O	0.48	134.07323	135.08051	25.425	[M+H]+1	117.0336 105.07003 91.05438 89.03870 79.05439
112	2,4-Dimethylbenzaldehyde	C9 H10 O	-1.65	134.07294	135.08051	22.459	[M+H]+1	117.03359 105.07004 91.05441 79.05441 77.03887
113	Naringenin	C15 H12 O5	-0.12	272.06844	271.0611	26.919	[M-H]-1	253.14461 225.14984 177.01927 151.00359 119.05008
114	Manninotriose	C18 H32 O16	0.5	504.16928	549.16748	2.318	[M+FA-H]-1	179.05688 119.03486 101.02432 89.02429 71.01375

115	Perillene	C10 H14 O	1.11	150.10463	151.11191	21.122	[M+H]+1	123.0442 109.06495 107.04920 95.04933 79.05441
116	6-Gingerol	C17 H26 O4	-0.38	294.183	293.17572	32.452	[M-H]-1	221.15448 236.10542 192.11522 177.09120 151.07570
117	Didymin	C28 H34 O14	0.44	594.19512	595.20239	20.432	[M+H]+1	433.11316 303.05002 287.05508 153.01831 127.03914
118	4-Oxoproline	C5 H7 N O3	-1	129.04246	128.03519	3.299	[M-H]-1	126.88091 57.03451 70.60007 82.02969 85.02924
119	Benzamide	C7 H7 N O	-2.5	121.05246	122.06026	1.532	[M+H]+1	105.04463 95.08571 77.03875 67.05455 65.03893

Table S2. The detailed information on 13 flavonoid components in SSF that enter the blood as prototypes.

Number	Name	Formula	Annot. DeltaMass [ppm]	Calc. MW	m/z	RT [min]	Reference Ion
1	4-Coumaric acid	C9 H8 O3	1.13	164.04753	165.05484	21.122	[M+H]+1
2	3',4'-Dihydroxyphenylacetone	C9 H10 O3	-1.06	166.06282	165.05554	17.674	[M-H]-1
3	Chlorogenic acid	C16 H18 O9	-0.02	354.09508	353.0878	17.519	[M-H]-1
4	Quinic acid	C7 H12 O6	-0.34	192.06332	191.05605	18.918	[M-H]-1
5	Quercetin-3β-D-glucoside	C21 H20 O12	1.04	464.09596	463.0885	21.71	[M-H]-1
6	Rutin	C27 H30 O16	0.75	610.15384	609.14643	21.351	[M-H]-1
7	Esculetin	C9 H6 O4	-0.59	178.0265	177.01923	18.379	[M-H]-1
8	Kaempferol	C15 H10 O6	0.22	286.0478	287.05518	22.553	[M+H]+1
9	Ferulic acid	C10 H10 O4	-0.73	194.05777	193.05049	23.574	[M-H]-1
10	Kaempferol-3-O-rutinoside	C27 H30 O15	0.51	594.15877	593.15149	22.092	[M-H]-1
11	Ethyl caffeate	C11 H12 O4	-0.65	208.07342	207.06615	23.689	[M-H]-1
12	Quercetin	C15 H10 O7	1.1	302.04299	303.05026	21.703	[M+H]+1
13	Afzelin	C21 H20 O10	0.11	432.1057	431.09842	23.604	[M-H]-1

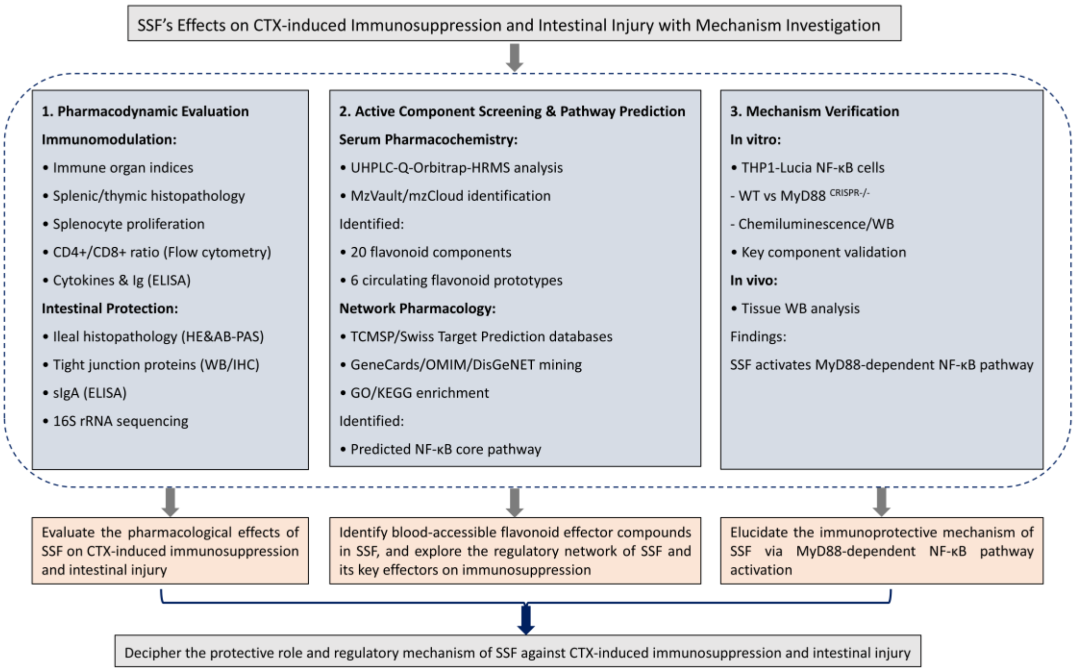

Figure S1. Schematic overview of the experimental workflow.

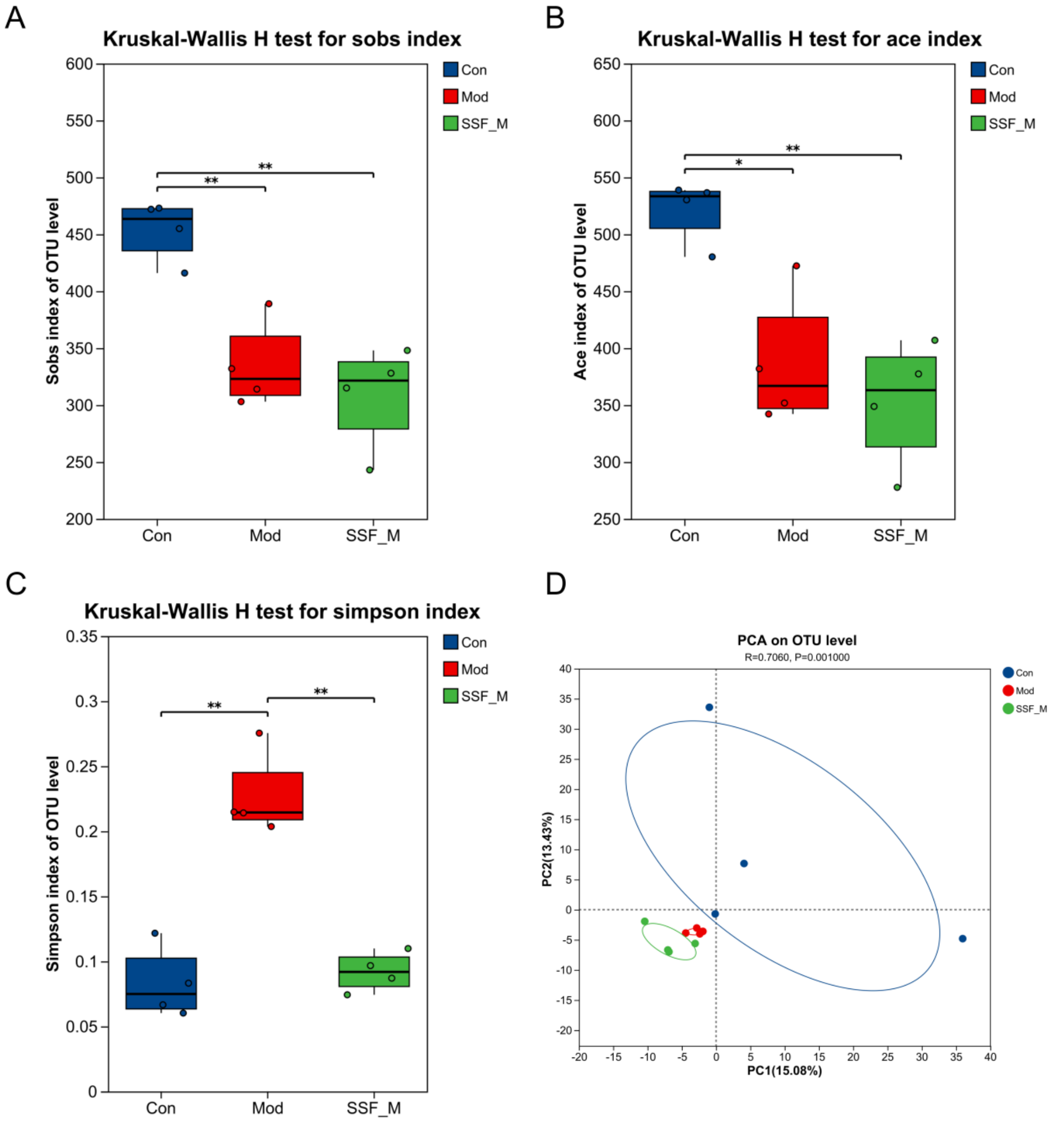

Figure S2. Changes in gut microbiota of immunocompromised mice with SSF treatment. Microbial richness indicated by Sobs (A), Ace (B), and Simpson indices (C). (D) Beta-diversity assay by principle component analysis (PCA).

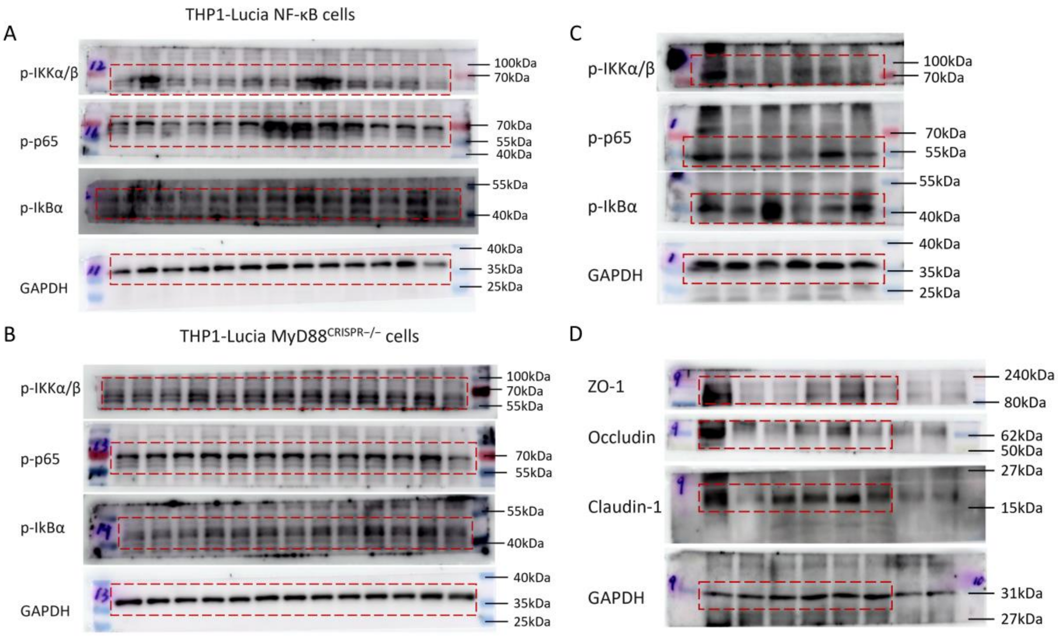

Figure S3. Immunoblotting raw images. Immunoblotting analysis of NF-κB pathway protein expression in WT (A) and MyD88CRISPR-/- (B) THP1-Lucia NF-κB cells stimulated with LPS and SSF for 0-24 h. (C) Protein expression of p-IKKa/β, IκBα, and p-NF-κB p65 in tissues of SSF-treated mice. (D) Immunoblotting of ZO-1, Occludin, and Claudin-1.
